# Evaluation of Potential Effects of NaCl and Sorbic Acid on Staphylococcal Enterotoxin A Formation

**DOI:** 10.3390/microorganisms3030551

**Published:** 2015-09-17

**Authors:** Nikoleta Zeaki, Peter Rådström, Jenny Schelin

**Affiliations:** Applied Microbiology, Department of Chemistry, Lund University, Lund 22100, Sweden; E-Mails: nikoleta.zeaki@tmb.lth.se (N.Z.); peter.radstrom@tmb.lth.se (P.R.)

**Keywords:** *Staphylococcus aureus*, enterotoxin A (SEA), staphylococcal food poisoning, food preservatives, prophage induction

## Abstract

The prophage-encoded staphylococcal enterotoxin A (SEA) is recognized as the main cause of staphylococcal food poisoning (SFP), a common foodborne intoxication disease, caused by *Staphylococcus aureus*. Studies on the production of SEA suggest that activation of the SOS response and subsequent prophage induction affect the regulation of the *sea* gene and the SEA produced, increasing the risk for SFP. The present study aims to evaluate the effect of NaCl and sorbic acid, in concentrations relevant to food production, on SOS response activation, prophage induction and SEA production. The impact of stress was initially evaluated on steady state cells for a homogenous cell response. NaCl 2% was found to activate the SOS response, *i.e*., *recA* expression, and trigger prophage induction, in a similar way as the phage-inducer mitomycin C. In contrast, sorbic acid decreased the pH of the culture to a level where prophage induction was probably suppressed, even when combined with NaCl stress. The impact of previous physiological state of the bacteria was also addressed on cells pre-exposed to NaCl, and was found to potentially affect cell response upon exposure to further stress. The results obtained highlight the possible SFP-related risks arising from the use of preservatives during food processing.

## 1. Introduction

Foodborne diseases have been a major public health concern for decades with annual reports of outbreaks from countries all around the world. *Staphylococcus aureus* is a notorious foodborne pathogen due to its ability to produce enterotoxins, staphylococcal enterotoxins (SEs), and therefore cause what is known as staphylococcal food poisoning (SFP), a common food-borne intoxication disease [1,2]. Staphylococcal enterotoxins have been the cause of 6.4% of food-borne outbreaks in the European Union (EU) in 2012, placing bacterial toxins as the third outbreak causative agent in the EU [3]. In USA, *S. aureus* was ranked as one of the five most frequent causes of food-borne outbreaks with more than 240,000 illnesses annually [4]. This reflects how staphylococcal outbreaks are not only a public health problem, but also an economical challenge for the social health system in developed countries.

*S. aureus* is a natural inhabitant of the nostrils, skin and hair of warm-blooded animals, including humans where 30%–50% of the population are carriers [2]. An important characteristic of the microorganism is that it can grow under a wide range of temperature, pH, and NaCl concentrations and thus the types of foods where *S. aureus* can be present vary from ready-to-eat meals to fermented foods [2,5]. The types of food most often connected with SFP are buffet meals and cheese products but there have also been incidents of intoxication from milk powder, chocolate milk, bakery products even ice-cream [3,6,7,8,9]. As main sources of contamination a poor hygiene of food handlers have been suggested, due to the ecological niche of the bacterium, along with non-hygienic processing equipment as well as inadequate cooling of foods which could allow growth and enterotoxin production by *S. aureus* [1].

The main source behind 80% of the SFP cases has been identified to be staphylococcal enterotoxin A (SEA) [1,10,11]. SEA is encoded by the *sea* gene located on the genome of temperate bacteriophages of the *Siphoviridae* family. Studies on *sea* gene regulation have shown that the life cycle of these phages has a significant role on the expression of the gene and on SEA production [12,13,14]. According to the related phage system of the λ phage, prophage induction is triggered by activation of the SOS response and results in increased levels of the phage replicative form copies (RF) in the cell. Hence, a leading hypothesis for the *sea*-carrying phages supports the link of the SOS response with the increase in RF copy levels intracellularly and subsequently the copies of the *sea* gene carried on the phage genome, which in turn would impact the SEA production levels.

The SOS response is one of the survival mechanisms activated when bacteria are exposed to adverse environments. It is essential for repair of DNA and stalled or collapsed replication forks and it can be triggered by a number of factors such as antimicrobial compounds, acids, heat and oxidative compounds, used during food processing [15,16]. Therefore, potential activation of the SOS response mechanism by the use of preservatives and its association with prophage induction and increased SEA production exposes the potential hazards in respect to current food processing methods and the risk for SFP.

The use of preservatives during food production is employed to control the presence of harmful bacteria, like pathogens, and create an environment that does not further favor their growth in the case of contamination during food processing [5]. Activation, though, of stress response mechanisms by bacteria after exposure to harsh environments, stimulates the expression of virulence related genes, like in the case of SOS response, in addition to increasing resistance of the cells to further stress. [15,17,18]. Therefore, the previous physiological state of cells is of critical importance for the effectiveness of preservation techniques, as it can affect the response of the pathogen towards the used preservatives. For example, in a study by Rodriguez-Caturla *et al.* [19], pre-incubation of *S. aureus* cells at low *a_w_* increased their survival capacity in cooked chicken breast stored at 10 °C. Cebrián *et al.* [20], demonstrated that *S. aureus* was able to increase its acid resistance if previously exposed to sub-lethal acidic pH.

In the present study, the hazards arising from the activation of the SOS response mechanism by the use of NaCl and sorbic acid (C_6_H_8_O_2_, E200) in concentrations relevant to food production, were explored in respect to the *sea* gene regulation and SEA production. The potential of prophage induction was investigated through the monitoring of the RF and *sea* gene copy levels, as well as the levels of SEA produced. Activation of the SOS response under the examined treatments was evaluated through the *recA* transcript levels. A strain previously shown to undergo prophage induction under the effect of mitomycin C (MMC), the inducible high-SEA producing Sa17, was used in the study. A continuous cultivation setup for the acquisition of Sa17 steady state cells was chosen to examine the effects of the two preservatives on a more homogenous cell population than that of batch cultivation. The impact of the previous physiological state of cells was further addressed, through the use of NaCl pre-stressed cells alongside cells grown under optimal conditions and cells in a steady state.

## 2. Experimental Section

### 2.1. Bacterial Strains and Cultivation Conditions

A food isolate of *S. aureus*, Sa17 (donated from SP Food and Bioscience, the former Swedish Institute for Food and Biotechnology, SIK, Göteborg, Sweden), previously characterized as an inducible high-SEA producing strain [14] was used for this study. Brain Heart Infusion (BHI) broth (Difco Laboratories, BD Diagnostic Systems, Le Pont de Claix, France), was the growth medium in all the experiments performed. Prior to inoculation, the cells were regenerated from a −80 °C glycerol stock, by streaking on BHI agar plates (Difco Laboratories, BD Diagnostic Systems) and incubation for 16 to 18 h (overnight) at 37 °C. A single colony was selected and sterilely transferred to fresh BHI broth for pre-culture preparation. For the batch cultivations a 50 mL falcon tube with 25 mL BHI or BHI supplemented with 2% NaCl was used, while for the bioreactor cultivations the pre-culture was prepared in a 100 mL flask with 50 mL BHI. The pre-cultures were grown overnight in a rotating incubator (New Brunswick Scientific, Innova 40/40R incubator Shakers, Eppendorf AG, Hamburg, Germany) at 37 °C and 200 rpm shaking.

#### 2.1.1. Bioreactor Setup

Adequate inoculum size for a starting optical density (OD) of 0.1 was calculated for 1 L working volume of BHI in a 2 L ADI 1010 Bio-Controller (Applikon, Schiedam, The Netherlands). Antifoam was used in the medium at final concentration of 0.5 mL·L^−1^ (Dow Corning Antifoam RD emulsion, VWR International Ltd., Poole, UK). The temperature was maintained at 37 °C; stirring was set at 500 rpm and the pH was kept constant at 7.4 by addition of 3 M KOH. Air was continuously sparged in the culture at 0.8 L·min^−1^ and was controlled by a mass flow meter (Bronkhorst Hi-Tech, Ruurlo, The Netherlands). A condenser with 5 °C cooling water was fitted to the bioreactor's headplate. Cells were grown in a batch mode until the point of glucose depletion when the bioreactor started to be fed with fresh medium, of identical composition to the batch start-up medium, and continuous cultivation was established. Steady state was assessed after at least five volume changes based on the criteria of constant CO_2_ production rates and constant OD values. When steady state was established, cells were harvested in batches of 100 mL, centrifuged immediately (3220× *g*, 5 min, 4 °C, 5810R table centrifuge, Eppendorf AG, Hamburg, Germany) and the pellets were re-suspended in (a) BHI + 2% NaCl, (b) BHI + 0.15% sorbic acid, (c) BHI + MMC at 1 mg·mL^−1^, (d) BHI + 2% NaCl + 0.15% sorbic acid and (e) BHI with no additives (optimal growth). A flask with 100 mL of cells straight from the bioreactor was used as a negative control for the quantification of the RF and *sea* gene copies while MMC as the positive control. All cultures (treatments a–e and controls) were grown as batch cultures and samples for OD, DNA, RNA and enzyme-linked immunosorbent assay (ELISA) analysis, were taken every hour for the next 4 h of incubation at 37 °C, 200 rpm shaking.

#### 2.1.2. Batch Culture Setup

**Figure 1:**
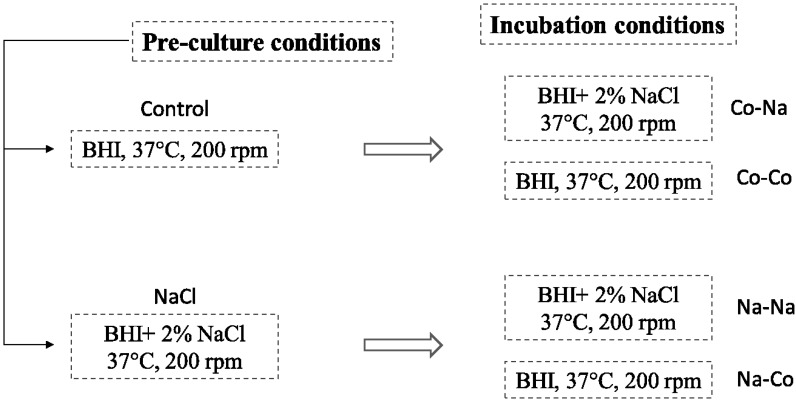
Schematic representation of the experimental set up of NaCl pre-stressed Sa17 cells and subsequent exposure to similar stress or optimal growth conditions. In total four different incubation conditions were investigated for *sea* gene regulation and SEA production.

To remove secreted metabolites and enterotoxins produced by *S. aureus* during overnight growth, pre-cultures were centrifuged (3220× *g*, 10 min, 4 °C, 5810R table centrifuge, Eppendorf AG, Hamburg, Germany) and cell pellets were washed twice in 25 mL of 0.9% sterile NaCl solution (Merck Millipore, Darmstadt, Germany) and re-suspended by vortexing in the same volume of 0.9% sterile NaCl solution. OD of the washed pre-cultures was measured at 620 nm (UV/Visible Spectrophotometer Ultrospec 2100 pro, GE Healthcare, Little Chalfont, UK) and the adequate inoculum size for a starting OD of 0.1 for 100 mL BHI or BHI supplemented with 2% NaCl (0.25 L baffled flask) was calculated. The various conditions for batch cultivation are shown in Figure 1. The incubation temperature for all four treatments was 37 °C and the rotation at 200 rpm. Samples were collected every 2 h until the eighth hour of incubation, for OD, DNA, and RNA isolation and ELISA analyses.

### 2.2. DNA Extraction

Purification of plasmid DNA was performed using the GeneJET Plasmid Miniprep Kit (Thermo Scientific, Waltham, MA, USA), as described in the producer’s protocol. Prior to extraction, cells were washed once with 0.9% NaCl solution. Concentration of extracted DNA (ng·μL^−1^) and purity (A_260/280_) were measured using BioDrop spectrophotometer (BioDrop TOUCH UV/Visible Spectrophotometer, Integrated Scientific Solutions Inc., Walnut Creek, CA, USA). All DNA samples were stored in −20 °C until further analysis.

### 2.3. Quantification of RF and sea Gene Copies

To follow genome copies of both bacteriophage RF and the *sea* gene, quantitative PCR (qPCR) analysis of the purified plasmid samples was performed. Specifically, a 20 μL qPCR reaction, including 4 μL of DNA template, was performed in a LightCycler 2.0 Carousel-based System® (Roche Diagnostics GmbH, Penzberg, Germany), using hybridization probes. The PCR mixture consisted of 1 × PCR *Tth* buffer, 3.25 mM MgCl_2_, 0.2 mM of dNTPs mixture, 0.5 μM of both forward and reverse primers, 0.03 μM of each hybridization probe and 0.05 U of *Tth* polymerase. All reagents mentioned above were purchased from Roche Diagnostics GmbH, apart from probes and primers, which were supplied by TIB Molbiol GmbH, Berlin, Germany (listed in Table 1). Autoclaved ultrapure water was used for all preparations and as negative control. The PCR protocol consisted of initial denaturation at 95 °C for 1 min, followed by 45 cycles of denaturation at 95 °C for 0 s (*i.e*., no hold at 95 °C), primer annealing at 46 °C for *sea* and 49 °C for RF, for 5 s, and extension at 72 °C for 25 s. A single fluorescence measurement was made at the end of the extension step. Cq values obtained were accepted when efficiency of amplification was between 1.8 and 2.0. Results are presented as the relative ratio between the reference sample (0 h sample) and the target sample according to the equation of Pfaffl (relative ratio = (*E*_target_)^Δ^^Cq target (control − sample)^) [21]. Primers used are listed in Table 1.

**Table 1:** Sequence of primers and probes with fluorescent dyes used for qPCR analyses of *sea* gene, replicative form of the bacteriophage genome (RF) and *rec*A transcripts.

Target	Primer/Probe	Sequence (5′ T 3′)
*sea* gene	ESA-1	5-ATGAGTTGGGCAAGATGGTT-3
	Tox A reverse	5-GGACTTGTTGTCCACGTTAGG-3
	Tox A-Fluo 1	CCTTTGGAAACGGTTAAAACGAATAAGAA-FL
	Tox A-Red 1	LC-R640-TGTAACTGTTCAGGAGTTGGATCTTCA p *
RF	forward	AAAATATAGCAATAACTACATCCG
	reverse	AAGTCCCTAAAAAGTCCCTA
	FL	ATGTTAAAAGTCTCCAGTTTGGATACA-FL
	LC	LC-R640-AgAAACCTTGTAACAACAGTATTTATTGGG p *
*rec*A gene	*rec*A forward	ATGCTCAAGCATTAGGCGTA
	*rec*A reverse	TTAGGTGTTAAAGCAGCAACTGA
	*rec*A FL	ACAAGGTCTTGAAATCGCCG-FL
	*rec*A LC	LC-R640-AGCATTTGTTAGAAGTGGTGCAGT-PH

* The acceptor probe is labeled with LC Red640 (LC-R640) at the 5′ end and the 3′ hydroxyl group is phosphorylated (p)

### 2.4. RNA Extraction

A modified version of the protocol described by Lövenklev *et al*. 2004 [22] was used for RNA extraction. In brief, cells from 5 mL culture were harvested by centrifugation at 4 °C, 3220× *g*. The resulting pellet was immediately frozen in liquid nitrogen and stored in −80 °C until extraction of RNA. For RNA extraction, each sample pellet was resuspended in 500 µL ice cold TES buffer, pH 7.5 (50 mM Tris (BDH Prolabo, VWR International, Stockholm, Sweden), 5 mM EDTA and 50 mM NaCl (Merck Millipore)) and the suspension was transferred to Precelly lysing kit tubes (VK 0.1) (Bertin Technologies, Montigny-le-Bretonneux, France). Cells were disrupted in a Precellys 24 unit (Bertin Technologies), in a three cycle run of 60 s at 6500 rpm. RNA was isolated using two steps of phenol-chloroform (600 μL:100 μL) extraction followed by one chloroform (600 μL) purification step. Precipitation of RNA was achieved in 0.1 vol of 3 M NaAc (pH 4.8) and 2.5 vol of 95% EtOH for at least for 1 h in −80 °C and further washed with 600 μL of 70% EtOH. The remaining traces of EtOH after expiration were removed by evaporation in room temperature, before the RNA was dissolved in 100 μL RNA storage solution (Ambion®, Thermo Fisher Scientific, Waltham, MA, USA). Concentration of extracted RNA (ng·µL^−1^) and purity (A_260/280_) were measured using BioDrop spectrophotometer (BioDrop TOUCH UV/Visible Spectrophotometer, Integrated Scientific Solutions Inc.). Prior to transformation into cDNA by reverse transcription, all RNA samples were treated with DNase to degrade contaminating traces of DNA. For this reaction, 15 μL of each RNA sample was mixed with 15 μL autoclaved diethyl pyrocarbonate (Fluka Analytical, Sigma-Aldrich, Buchs SG, Switzerland) treated water, 15U RQ1 RNase-free DNase (Promega Co., Madison, WI, USA) and 1× reaction buffer. The suspension was incubated at 37 °C for 45 min. Stop solution, in combination with incubation at 65 °C for 10 min, was further added to inactivate the DNase. All samples were stored at −80 °C until further analysis.

### 2.5. cDNA Synthesis

First-strand cDNA was synthesized in two separate reverse-transcription reactions using reverse primer specific *recA* transcripts (Table 1). The reactions were performed with a Gene Amp 9700 thermal cycler (Perkin-Elmer, Waltham, MA, USA). The total volume of the mixture was 20 μL and contained 0.5 μg of total RNA, the reverse primer (TIB Molbiol GmbH, Berlin, Germany) at a concentration of 0.5 mM, each deoxynucleoside triphosphate (dATP, dTTP, dCTP, and dGTP; Roche Diagnostics GmbH) at a concentration of 5 mM, 20 U of RNasin RNase inhibitor (Promega Co., Madison, WI, USA), 5 mM dithiothreitol 1× first-strand buffer, and 200 U of Superscript II RNase H^−^ reverse transcriptase (Invitrogen, Thermo Fisher Scientific, Waltham, MA, USA). Autoclaved diethyl pyrocarbonate-treated water (ddH_2_O) was used both in the reaction mixture and to dilute mRNA samples. Before RT enzyme was added, the reaction mixture was heated to 65 °C for 5 min and then chilled on ice. After a brief centrifugation and addition of the RT enzymes, the reaction mixture was incubated at 42 °C for 50 min, and the reaction was terminated by incubation at 70 °C for 15 min. The cDNA was diluted 10-fold in autoclaved ddH_2_O before qPCR analysis. The qPCR protocol was similar as the one described above with only difference in the primer annealing temperature that was 55 °C.

### 2.6. ELISA

ELISA was performed according to the revised laboratory protocol for staphylococcal enterotoxin A, described before by Wallin-Carlquist *et al*. 2010 [23]. Absorbance was measured at 405 nm with Multiskan Ascent® spectrophotometer (Thermo Fisher Scientific). Obtained absorbance values were plotted against toxin concentrations. Absorbance values for the standard samples were plotted against the known concentrations of SEA and a standard curve was created. The concentrations of the unknown samples were calculated using the linear regression and expressed in ng·mL^−1^ of toxin.

## 3. Results and Discussion

### 3.1. Response of Sa17 Steady State Cells to NaCl and Sorbic Acid

**Figure 2:**
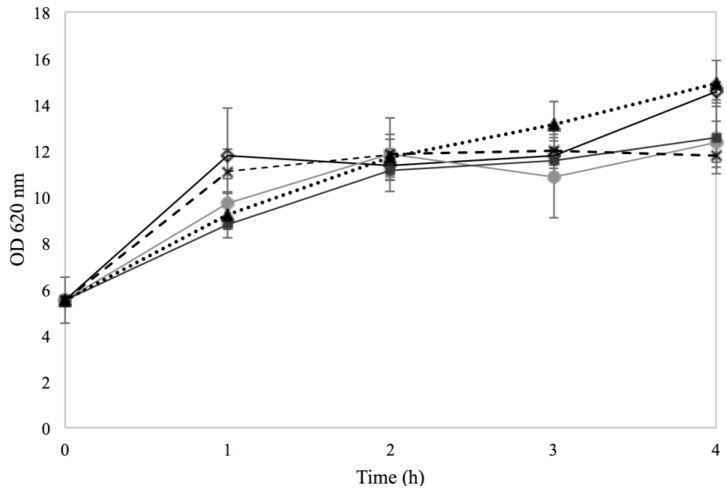
Growth of Sa17 steady state cells under exposure to 2% NaCl (◊, solid black line), 0.15% sorbic acid (●, solid light grey line), 2% NaCl + 0.15% sorbic acid (■, solid dark grey line) in batch cultivation conditions. Controls with MMC (**×**, dashed line) and BHI with no additives (▲, dotted line) were included. Results are obtained from two independent experiments. The *Y* axis represents OD measured at 620 nm and the *X* axis the time in hours. Time point 0 h represents the moment when the steady state cells (OD 5.5) were harvested, transferred into flasks and subjected to the various treatments.

Regarding growth of Sa17 the presence of 0.15% sorbic acid and the combination of 2% NaCl and 0.15% sorbic acid somewhat reduced growth initially but overall no major differences could be observed between the different treatments (Figure 2). The lower pH (5–5.5) in cultures with sorbic acid compared to cultures with NaCl (6–6.5) is most likely the reason for this minor initial reduction. By the fourth hour of incubation, cells grown in the presence of 2% NaCl and in BHI with no additives, appeared to reach the highest OD compared to the other treatments. *S. aureus* is known to be a highly osmo-tolerant microorganism, and the obtained results confirm its ability to grow as efficiently under the used NaCl concentrations, as in BHI with no additives [2].

**Figure 3:**
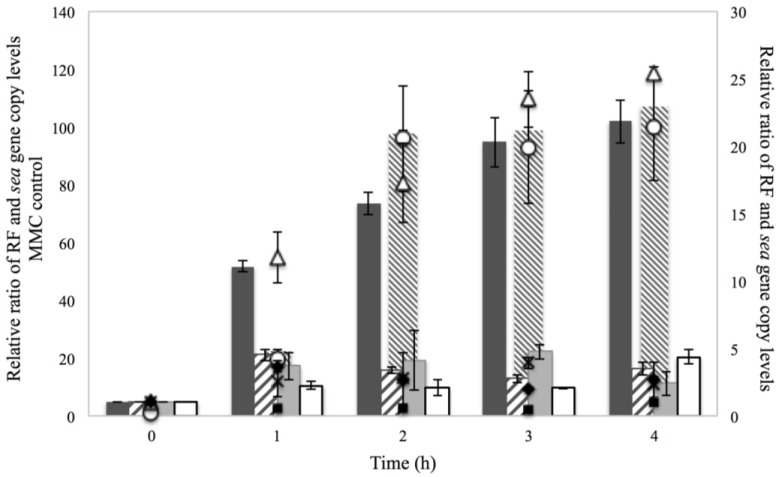
RF (bars) and *sea* gene (symbols) copies of Sa17 steady state cells under exposure in batch conditions to 2% NaCl (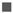) and (Δ), 0.15% sorbic acid (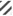) and (♦), 2% NaCl + 0.15% sorbic acid (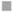) and (**×**) and the controls of MMC (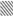) and (○) and BHI with no additives (□) and (■), obtained from two independent experiments. The left *Y* axis represents the relative ratio levels, as calculated from Cq values, of RF and *sea* gene for MMC control while the right *Y* axis represents the relative ratio of RF and *sea* gene for NaCl, sorbic acid, NaCl + sorbic acid and BHI with no additives. The *X* axis represents the time in hour.

Investigation of the RF and *sea* gene copies to assess the impact of the treatments on prophage induction and *sea* gene regulation is demonstrated in Figure 3. Interestingly, it was observed that 2% NaCl increased the levels of RF and *sea* gene copies, in a pattern similar to that of the positive control with MMC. Although a less pronounced impact of NaCl compared to that of MMC was obtained, the results indicated that the investigated concentration could trigger prophage induction in an analogous manner as previously observed for the phage-inducing agent [13,14]. This is further supported by the observation that in the control condition of BHI with no additives, the RF and *sea* gene copy levels remained low throughout the four hours of incubation. The impact of NaCl on prophage induction has also been observed in a study by Harris *et al.* [24], where NaCl concentrations relevant to meat production were investigated for their effect on the levels of the prophage-encoded Shiga toxins of *E. coli* O157:H7. It was demonstrated that 2% NaCl triggered prophage induction and increased the levels of *stx2A* transcripts and *stx2A* DNA abundance, leading to enhanced toxin production.

In contrast to NaCl, the use of 0.15% sorbic acid on Sa17 steady state cells had little impact on the levels of RF and *sea* gene copies. The relative ratios for both RF and *sea* gene remained below five and was at the most in total about seven folds lower than the levels observed from 2% NaCl (Figure 3). Similar levels were detected for the combination of 2% NaCl and 0.15% sorbic acid. This finding was interesting as it appeared that use of sorbic acid suppressed the effect of NaCl on prophage induction observed earlier.

The absence of effect of sorbic acid on the phage was remarkable as it is known that weak acids can activate the SOS response mechanism and therefore prophage induction would be expected to occur [16]. In a study by Wallin-Carlquist *et al.* [23] acetic acid was related to prophage induction as it was observed that it boosted *sea* expression in the *S. aureus* strains, Mu50 and Sa45, at pH 6 and 5.5 respectively. The low levels of RF and *sea* gene copies observed in the present study could be attributed to the low pH, close to 5, that characterized the respective cultures. The antimicrobial activity of weak acids lays on their general ability to lower both the extracellular pH in addition to permeating the cell membrane and decreasing the intracellular pH with subsequent inhibition of essential metabolic and anabolic processes [15]. Therefore at pH 5, the cell could stimulate most of its energy towards re-establishing its homeostasis and thus other processes including protein synthesis and activation of response systems would be stalled. Weinrick *et al.* [25], in an investigation regarding the effects of mild acid on gene expression in *S. aureus*, observed that at pH 5.5 the majority of phage-encoded genes were down-regulated compared to pH 7.5.

The physiological state of cells at the time of exposure to a stress factor, and the stress conditions previously encountered by the cell, are known to affect the response of an organism towards further stress imposed. In this study, cells entered stationary phase after the first hour of incubation under the batch conditions, and the response to sorbic acid could therefore be less pronounced than in the case of exponentially growing cells. In the study of Davis *et al.* [26], on acid tolerance of *Listeria monocytogenes*, it was shown that resistance against exposure to acid stress was dependent on the growth phase of the cells, with those being in stationary phase showing increased tolerance to lethal pH (pH 3.0) compared to exponentially growing cells. Additionally, the cells in the present study originated from a continuous cultivation, where their metabolism was stimulated towards maintaining a steady growth rate for a number of generations before they were exposed to NaCl and sorbic acid. It could hence be possible that maintenance of balanced growth and metabolism has induced certain adaptive responses in the cell, which became more resistant towards certain stresses. Another explanation could be that the cells, due to their previous growth in a continuous mode, would need more time to respond or adapt to acid stress, since the latter is known to be harsh for bacterial cells.

### 3.2. Activation of the SOS Response

**Figure 4:**
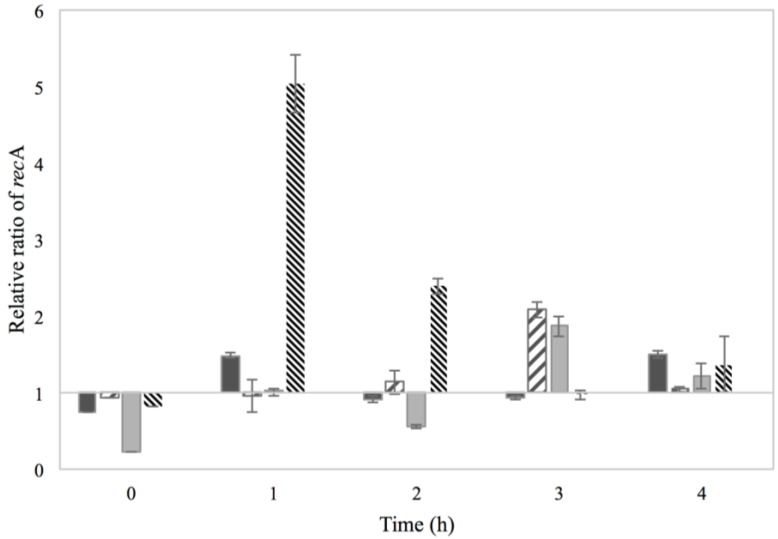
Relative *rec*A mRNA transcript levels of Sa17 steady state cells under exposure in batch conditions to 2% NaCl (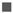), 0.15% sorbic acid (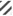), 2% NaCl + 0.15% sorbic acid (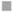) and the control of MMC (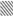), obtained from two independent experiments. The *Y* axis represents the *rec*A relative ratio levels as calculated from Cq values and the *X* axis the time in h. Values below/above 1 in relative ratio demonstrate down- or up-regulation of *rec*A in relation to the control condition of BHI with no additives (value of 1).

The activation of the SOS response mechanism due to the use of 2% NaCl and/or 0.15% sorbic acid was investigated through the transcripts of *rec*A produced. The *rec*A mRNA transcripts of each treatment were normalized to those of BHI with no additives and the calculated *rec*A relative ratios are presented in Figure 4.

A modest increase in the *rec*A transcript levels was noted for 2% NaCl cultures during the first and fourth hour of incubation, reaching the level of around 1.5 in relative transcript ratio. The observed transcription pattern could indicate activation of the SOS response in two phases, either due to a latter activated subpopulation or due to the increasing intracellular accumulation of solutes over time. An interesting observation was noted for the cultures treated with 0.15% sorbic acid and the combination of sorbic acid and NaCl, where increased *rec*A transcript levels, about two in relative ratio, were observed after 3 h of incubation. Though these treatments had no evident effect on the RF and *sea* gene levels, it appears that SOS activation did actually occur. The low pH of the culture however that affects enzyme activity and protein synthesis could be the parameter influencing the production of SEA. Increased *rec*A transcript levels were observed for MMC treatment during the first hour of incubation, which was expected considering the mechanism of action of this agent.

### 3.3. SEA production

**Figure 5:**
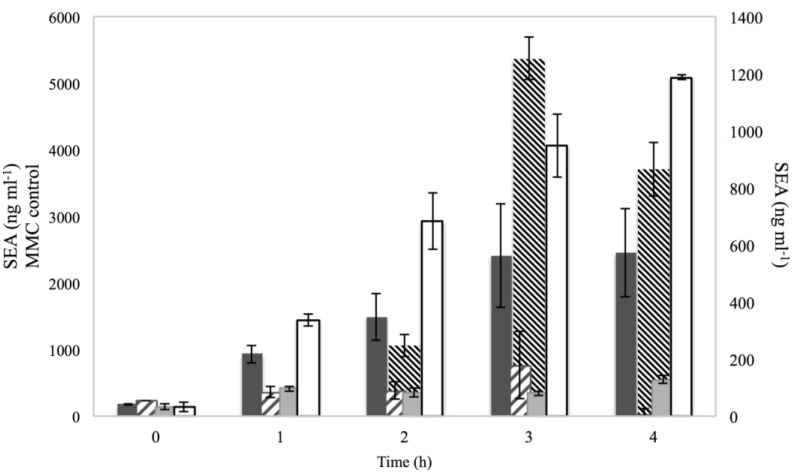
SEA levels produced by Sa17 steady state cells under exposure in batch conditions to 2% NaCl (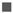), 0.15% sorbic acid (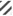), 2% NaCl + 0.15% sorbic acid (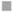) and the controls of MMC (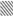) and BHI with no additives (□), obtained from two independent experiments. The left *Y* axis represents the SEA in ng·ml^−1^ for MMC control, while the right *Y* axis represents the SEA in ng·ml^−1^ for NaCl, sorbic acid, NaCl + sorbic acid and BHI with no additives. The *X* axis represents the time in h.

The impact of the different treatments on SEA production was also evaluated and is presented in Figure 5.

For 2% NaCl, the SEA levels produced did not correspond to the observations on RF and *sea* gene copy levels, since the expected boost of SEA formation was not seen, as in the case of the control with MMC. Overall SEA formation from cultures with 2% NaCl did not exceed 800 ng·mL^−1^ (peak at 3 h of incubation), which was below the level of SEA produced under BHI with no additives at the same point of incubation. This difference between the copies of the gene and the SEA produced could be attributed to the suboptimal NaCl concentration for enterotoxin production as well as the state of the cell under osmotic stress. To survive osmotic stress, bacteria are known to accumulate compatible solutes that help them maintain the cytoplasmic osmotic pressure higher than that of the environment. During this maintenance energy state, it is possible that biosynthesis and secretion of exoproteins are down regulated [27]. Among the solutes accumulated by *S. aureus* under NaCl stress are glycine betaine and proline [28]. These amino acids are also important for SEA synthesis and their absence has been shown to have a negative effect on the yield (%) of the enterotoxin [29]. For the treatments of 0.15% sorbic acid and combination of NaCl and sorbic acid, the levels of SEA produced were in agreement with what was detected for RF and *sea* gene copies, as they remained below 200 ng·mL^−1^, which was lower than the SEA produced under BHI with no additives.

### 3.4. The Impact of Physiological Cell History on Prophage Induction

To investigate the impact of the previous physiological state of cells upon subsequent exposure to stress in respect to prophage induction and *sea* gene regulation, Sa17 cells were grown overnight (16–18 h) in BHI under optimal conditions (control pre-culture) and BHI + 2% NaCl (NaCl pre-culture), and the resulting pre-cultures were used to inoculate flasks with BHI + 2% NaCl and BHI with no additives (Figure 1). The effect on growth, RF and *sea* gene copy levels, the *rec*A transcript levels and the SEA produced was investigated under batch cultivation conditions. The results presented derive from one experiment, and more replicates are required to further support the obtained observations.

**Figure 6:**
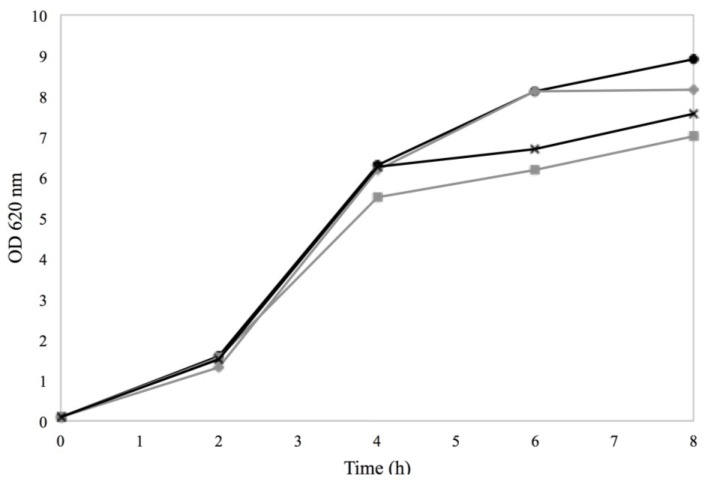
Growth in batch cultivation of Sa17 cells under exposure to either NaCl stress or optimal conditions. The cells were pre-grown under optimal or NaCl stress. The *Y* axis represents OD measured at 620 nm and the *X* axis the time in hours. The different treatments investigated are designated with different symbols. Co–Co is presented with (●), Co–Na with (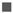), Na–Co with (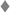) and Na–Na with (**×**). The results are obtained from one experiment.

Regarding growth of Sa17 and irrespective to the pre-culture conditions, when cells were exposed to BHI + 2% NaCl (Co–Na, Na–Na), slightly slower growth was observed compared to the cultures growing in BHI with no further additives (Co–Co, Na–Co) (Figure 6). The difference in the growth pattern was noted after the fourth hour of incubation. Yet, Sa17 cells originating from the control pre-culture and further grown in BHI + 2% NaCl (Co–Na), were those exhibiting the lowest growth. It can be therefore presumed that pre-cultivation in the presence of NaCl triggered an adaptive response of the *S. aureus* cells which were less affected by subsequent exposure to stress compared to optimally pre-grown cells.

**Figure 7:**
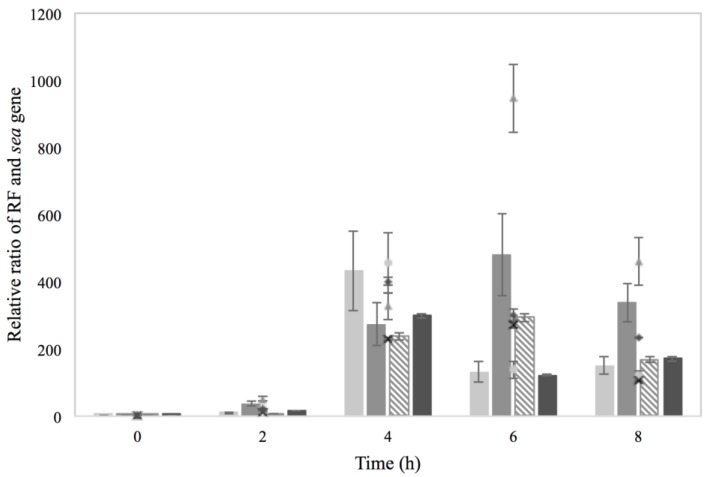
RF (bars) and *sea* gene (symbols) copies formed in batch cultivation of Sa17 cells under exposure to either NaCl stress or optimal conditions. The cells were pre-grown under optimal or NaCl stress. The *Y* axis represents the relative ratio levels of RF and *sea* gene as calculated from Cq values obtained from one independent experiment and the *X* axis represents the time in h. The investigated treatments are designated: Co–Co with (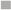) and (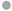), Co–Na with (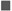) and (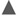), Na–Co with (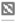) and (**×**) and Na–Na with (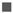) and (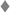).

Investigation of the RF and *sea* gene copy levels showed a relatively similar increase, for the studied conditions, at the fourth hour of growth, which then dropped for all treatments apart from that of Co–Na (Figure 7). Interestingly, the latter exhibited a slight increase on both RF and *sea* gene copy levels between the sixth and eighth hour of incubation. Considering the observations on growth of Sa17 under the condition of Co–Na, it can be concluded that the impact of osmotic stress, regarding prophage induction, is greater on previously optimally grown cells than in cells pre-exposed to NaCl.

Quantification of the SEA levels formed under the four tested conditions, did not reveal a clear enough pattern for any comparative conclusions to be drawn (data not shown). Nevertheless, of interest was the observation on the SEA levels formed under the Na–Na culture condition, where increased enterotoxin levels were observed at the fourth hour of incubation, compared to the other treatments.

**Figure 8:**
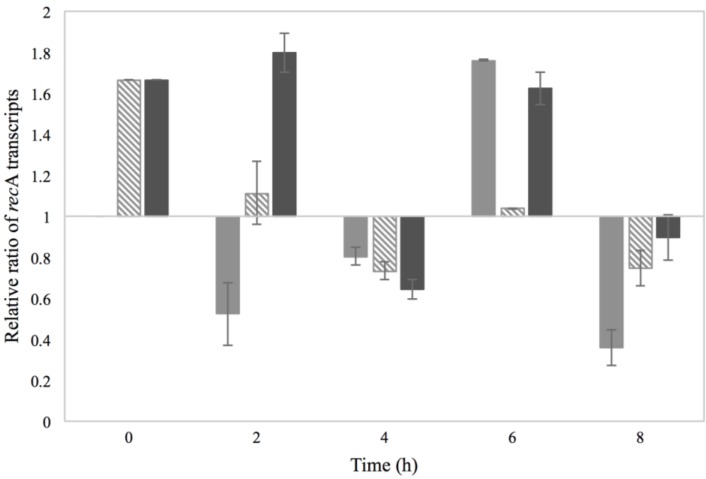
Relative *rec*A mRNA transcript levels formed in batch cultivation of Sa17 cells under exposure to either NaCl stress or optimal conditions. The cells were pre-grown under optimal and NaCl stress. The *Y* axis represents the *rec*A relative ratio levels in reference to the condition Co–Co, as calculated from Cq values obtained from one independent experiment, and the *X* axis represents the time in h. The different growth conditions are designated: Co–Na (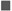), Na–Co (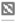) and Na–Na (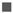).

The impact of the previous physiological state of cells on prophage induction was investigated on the *rec*A transcripts to examine possible activation of the SOS response. The detected *rec*A transcripts from each treatment were normalized to those of Co–Co (control conditions for both pre-culture and main culture growth) and the calculated relative ratios are demonstrated in Figure 8. As observed, pre-exposure of cells to NaCl resulted in increased *rec*A transcript levels from the beginning of incubation; an observation that implies already enhanced *rec*A transcription from the pre-culture environment. However, in the progression of growth only the Na–Na cultures maintained the enhanced *rec*A transcription levels, indicating that the cells managed to recover when the conditions of the environment became favorable. For the Co–Na cultures, an increase on the levels of *rec*A transcripts was observed at the sixth h of incubation, which corresponds time wise with the increase on the levels of RF and *sea* gene copies.

From the results obtained we can conclude that cells pre-exposed to stress were less affected by subsequent exposure to similar stress regarding *sea* regulation and SEA production, compared to cells previously grown under optimal conditions. A more extensive study would provide more insights on the impact of cell history on subsequent exposure to stress and response in terms of for example SEA production. Investigation of not only osmotic but also other stresses encountered in food production should be included for a complete factorial design construction that will greatly implement the presented observations.

## 4. Conclusions

In this study we have shown that use of NaCl as a food preservative could potentially increase the risk for SFP, as it triggers phage induction and increase the levels of *sea* gene produced by the inducible high SEA-producing Sa17. Further it was shown that pH is an important parameter in regard to SEA production as at levels around pH 5 the effect of sorbic acid on phage induction was suppressed. The impact of cell history also appeared to have an important role on the response towards imposed stress. The use of preservatives in food processing is one of the most established ways to ensure safe food products. In light of the newly acquired knowledge on *sea* gene regulation and influence of the phage life cycle, more studies investigating the effects of preservation methods on the regulation of SEA production are needed to evaluate possible risks during food processing.
